# TGF-β1 Downregulates the Expression of CX_3_CR1 by Inducing miR-27a-5p in Primary Human NK Cells

**DOI:** 10.3389/fimmu.2017.00868

**Published:** 2017-07-25

**Authors:** Stefano Regis, Fabio Caliendo, Alessandra Dondero, Beatrice Casu, Filomena Romano, Fabrizio Loiacono, Alessandro Moretta, Cristina Bottino, Roberta Castriconi

**Affiliations:** ^1^Dipartimento di Ricerca e Diagnostica, Istituto Giannina Gaslini, Genova, Italy; ^2^Dipartimento di Medicina Sperimentale, Università degli Studi di Genova, Genova, Italy; ^3^Dipartimento delle Terapie Oncologiche Integrate, Ospedale Policlinico San Martino IRCCS, Genova, Italy; ^4^Centro di Eccellenza per la Ricerca Biomedica, Genova, Italy

**Keywords:** TGF-β1, chemokine receptors, microRNAs, NK cells, tumor microenvironment

## Abstract

Activity of human natural killer (NK) cells against cancer cells is deeply suppressed by TGF-β1, an immunomodulatory cytokine that is released and activated in the tumor microenvironment. Moreover, our previous data showed that TGF-β1 modifies the chemokine receptor repertoire of NK cells. In particular, it decreases the expression of CX_3_CR1 that drives these effectors toward peripheral tissues, including tumor sites. To identify possible mechanisms mediating chemokine receptors modulation, we analyzed the microRNA profile of TGF-β1-treated primary NK cells. The analysis pointed out miR-27a-5p as a possible modulator of CX_3_CR1. We demonstrated the functional interaction of miR-27a-5p with the 3′ untranslated region (3′UTR) of CX_3_CR1 mRNA by two different experimental approaches: by the use of a luciferase assay based on a reporter construct containing the CX_3_CR1 3′UTR and by transfection of primary NK cells with a miR-27a-5p inhibitor. We also showed that the TGF-β1-mediated increase of miR-27a-5p expression is a consequence of miR-23a-27a-24-2 cluster induction. Moreover, we demonstrated that miR-27a-5p downregulates the surface expression of CX_3_CR1. Finally, we showed that neuroblastoma cells induced in resting NK cells a downregulation of the CX_3_CR1 expression that was paralleled by a significant increase of miR-27a-5p expression. Therefore, the present study highlights miR-27a-5p as a pivotal TGF-β1-induced regulator of CX_3_CR1 expression.

## Introduction

Human natural killer (NK) cells mainly consist of CD56^bright^ CD16^low/neg^ KIR^neg^ perforin^low^ and CD56^dim^ CD16^pos^ KIR^pos^ perforin^high^ cells, which represent sequential stages of maturation and NK subsets with different function and tissue distribution (1, 2). CD56^bright^ NKs, which release large amount of soluble factors (IFN-γ, GM-CSF, and TNF-α) in response to proinflammatory cytokines, are poorly represented in peripheral blood, while populating most tissues, and, in particular, secondary lymphoid organs (SLOs) (3, 4). Conversely, CD56^dim^ cells, which are highly cytotoxic effectors, represent the largest percentage of circulating NKs, while being detectable only in selected peripheral tissues such as lung and breast (1). Notably, upon appropriate stimulation, CD56^dim^ cells increase the cytolytic potential, become cytokines producers, and also acquire the capability to migrate to SLO (5, 6).

The NK cells distribution in physiological and pathological conditions is dictated by the expression of chemokine receptors that retain or drive the migration toward tissues of one or another NK cell subset. Peripheral blood (resting) CD56^dim^ and CD56^bright^ NK cells share the expression of CXCR4 (CXCL12 receptor) (7) and CXCR3 (CXCL4, 9, 10, 11 receptor). CD56^bright^ NKs selectively express CCR5 (CCL3-5 receptor) and CCR7 that guides their migration toward tissues with high levels of CCL19 and CCL21, such as SLO. Conversely, the expression of CXCR1 (CXCL8 receptor), CXCR2 (CXCL1, 2, 3, 5, 8 receptor), and CX_3_CR1 (CX_3_CL1 receptor) is restricted to CD56^dim^ NKs (4), which are also characterized by the peculiar expression of the ChemR23 specific for chemerin (8).

CX_3_CL1, also termed fractalkine, is the only known member of the CX_3_C chemokine family. It is synthesized as a membrane anchored molecule expressed by cells such as endothelial cells, epithelial cells, osteoblasts, and mesenchymal stromal cells. CX_3_CL1 can be shed by A Disintegrin And Metalloproteinases (ADAM) 10 or 17 in non-inflammatory and inflammatory conditions, respectively (9). Once released, it interacts with the specific receptor (CX_3_CR1), a molecule belonging to the 7-transmembrane G protein-coupled receptor family, which is expressed by different immune cell types, including monocytes, dendritic cells (DCs), T, and NK lymphocytes.

Tumor tissues set up different strategies to escape immune recognition and in particular to affect chemokine/chemokine receptor axes such as CX_3_CL1/CX_3_CR1, which are crucial for the recruitment of cells such as CD56^dim^ NK cells exerting tumor suppressive properties. A possible strategy is to unbalance in the tumor microenvironment the equilibrium between CD56^dim^ and CD56^bright^ attracting chemokines. Immature, poor cytolytic CD56^bright^ cells represent the major NK subset present in most tumor tissues (4, 10), and breast carcinomas have been shown to decrease the expression of CXCL2 and CX_3_CL1 while increasing that of CCL5 and CCL19 (1). Accordingly, CX_3_CL1 expression in breast carcinoma specimens was shown to correlate with a good prognosis (11).

Another possible mechanism exerted by tumors for limiting immune surveillance is represented by their capability of modifying the chemokine receptor repertoire of crucial effector cells, thus affecting their responsiveness to given chemoattractant gradients. Among the soluble mediators involved, TGF-β1 shows a pivotal role in chemokine receptors modulation (12). TGF-β1 is pleiotropic cytokine whose major physiologic role is to limit the duration of immune responses and promote tissue repair (13, 14). In the tumor microenvironment, its release and activation by cancer cells and immune cells, including tumor-associated macrophages (TAM), is exacerbated (9, 15). This results in tumor-promoting side effects including angiogenesis and suppression of anticancer immunity. In NK cells, tumor-derived and recombinant TGF-β1 has been shown to downregulate the expression of NKp30 and NKG2D activating receptors involved in tumor recognition and DC editing (16, 17) and to modulate the chemokine receptor repertoire. In particular, TGF-β1 increases the expression of CXCR3 and CXCR4 (12, 18), involved in peripheral tissues and bone marrow (BM) recruitment, respectively, and decreases that of CX_3_CR1, which drives effector cells toward peripheral tissues, including central nervous system (CNS) (19–21). Interestingly, in glioma cells TGF-β1 has also been shown to downregulate CX_3_CL1 (22). The presence of CD56^dim^ NK cells, characterized by low expression of CX_3_CR1, has been detected in ascitic fluids of ovarian carcinoma patients (10). Moreover, a possible systemic effect has been demonstrated in patients with high-risk (stage 4 or M) neuroblastoma (NB), who were characterized by the presence of unconventional CD56^dim^ CX_3_CR1^low^ NKs, both in metastatic sites, such as BM and peripheral blood (12).

Understanding how TGF-β1 impairs the expression of CX_3_CR1 in CD56^dim^ NK has potential clinical implications, since it could improve the current knowledge of the mechanisms that regulate the immune responses and, in cancer, participate in the escape from immune surveillance. MicroRNAs (miRNAs), small non-coding RNA molecules, are key players in the regulation of gene expression and modulate several biological processes, including immune responses. In this study, we performed a profile of NKs to identify miRNA that could play roles in TGF-β1-mediated modulation of chemokine receptors expression. We identified miR-27a-5p as a negative regulator of CX_3_CR1 expression.

## Materials and Methods

### NK Cells Purification

NK cells were purified from peripheral blood mononuclear cells of healthy donors using the Human NK Cell Isolation kit (Miltenyi Biotec) (12). A total of 13 unrelated healthy donors were used for all the experiments described. Donors provided an informed consent according to the procedures approved by the Ethics Committee of Ospedale Policlinico San Martino (39/2012).

### NK Cell Treatments for miRNA and mRNA Expression Analysis

To obtain miRNA profiling, NK cells were incubated for 24 h in the presence of complete medium (RPMI 1640 with 10% of fetal bovine serum, 2 mM glutamine, 50 mg/mL penicillin and 50 mg/mL streptomycin) supplemented with TGF-β1 at the final concentration of 40 or 5 ng/mL. Control cells were represented by NK cells cultured in the presence of complete medium alone (12). For miRNA validation and mRNA expression analysis, NK cells were incubated in the presence of 40 or 5 ng/mL of TGF-β1 or complete medium for 12 or 24 h.

### RNA Isolation

RNA containing the small RNA fraction was extracted using the miRCURY RNA Isolation Kit—Cell and Plant (Exiqon) according to the manufacturer guidelines. RNA amount was determined using the Quant-iT RiboGreen RNA Assay Kit (Invitrogen) following to the manufacturer instructions.

### miRNA Profiling

miRNA profiling was performed according to the TaqMan MicroRNA Array Workflow (Applied Biosystems). Briefly, 100 ng of RNA comprehensive of the small RNA fraction were reverse transcribed using the TaqMan MicroRNA Reverse Transcription kit with the Megaplex RT Primers Human Pool A + B set v3.0 (Applied Biosystems). A preamplification step was performed using the TaqMan PreAmp Master Mix with the Megaplex PreAmp Primers Human Pool A + B set v.3.0 (Applied Biosystems). Preamplified cDNAs were loaded in TaqMan Array Human MicroRNA Cards A and B set v3.0 (Applied Biosystems). Amplification reactions were performed using an Applied Biosystems ViiA 7 Real-Time PCR System. Data were analyzed using the miScript miRNA PCR Array Data Analysis Tool.^1^ miRNAs expression was normalized to the U6 snRNA expression. Data from this miRNA profiling have been submitted to the NCBI Gene Expression Omnibus^2^ under accession no. GSE98769. miRNAs that showed a fold change >2 or <0.5 between TGF-β1-treated and -untreated NK cells in three different donors were considered to be differentially expressed. Using the computational prediction on-line tools TargetScan^3^ (23), miRanda^4^ (24), and miRmap^5^ (25), selected miRNAs were investigated as possible regulators of CXCR4, CXCR3, and CX_3_CR1 expression.

### Expression of miRNAs

Expression of selected miRNAs was evaluated using TaqMan MicroRNA Assays as described by the manufacturer (Applied Biosystems). Briefly, 10 ng of RNA were reverse transcribed using the TaqMan MicroRNA Reverse Transcription kit primed with the specific RT primers. Real-time PCR was performed in quadruplicate using the specific primers. Expression of each miRNA was normalized to the RNU44 expression.

### Expression of CX_3_CR1 mRNA

To evaluate the expression of CX_3_CR1 mRNAs, 100 ng of RNA were reverse transcribed using the SuperScript VILO cDNA Synthesis Kit (Invitrogen). The cDNA was used for real-time PCR using the specific primers contained in the TaqMan Gene Expression Assay (Applied Biosystems). CX_3_CR1 expression was normalized to the GAPDH expression. Experiments were performed in quadruplicate.

### CX_3_CR1 3′ Untranslated Region Construct

A 700 bp fragment of the CX_3_CR1 3′ untraslated region (3′UTR) containing the putative target site for miR-27a-5p was amplified by PCR using primers CX3UTR2F and CX3UTR2R, containing the NheI and XhoI sites in their 5′ ends, respectively. The PCR product was purified using the MinElute PCR Purification Kit (Qiagen), NheI-XhoI digested and ligated to the NheI-XhoI digested pmirGLO vector (Promega) using the Rapid DNA Ligation kit (Roche, Basel, Switzerland). Ligation product was used for transformation of TOP10 *Escherichia coli* competent cells (Invitrogen). Colonies containing the recombinant plasmid were selected by PCR using primers CX3UTR2F/CX3UTR2R. Plasmid DNA was purified using the Illustra PlasmidPrep Mini Spin Kit (GE Healthcare). The inserted fragment and flanking sequences were sequenced using primers CX3UTR2F, CX3UTR2R, CX3UTR22R2, and CX3UTR22F2. Sequencing was performed using the BigDye Terminator v3.1 Cycle Sequencing Kit (Applied Biosystems) in a 3100 Genetic Analyzer (Applied Biosystems). The selected plasmid is reported as pmirCX3UTRWT. Primers, when not otherwise indicated, were designed by primerBLAST.^6^ Primer sequences are reported in Figure S1 in Supplementary Material.

### Site-Directed Mutagenesis

A mutated version of pmirCX3UTRWT (pmirCX3UTRMT), containing a C>G mutation in the putative target site for miR-27a-5p, was prepared by site-directed mutagenesis using the Geneart Site-Directed Mutagenesis System (Invitrogen) according to the manufacturer guidelines. Oligonucleotides (CX3MUTF and CX3MUTR) were designed using the QuickChange Primer Design on-line tool (Agilent Technologies^7^). Presence of the mutation was confirmed by restriction analysis and by sequencing the mutagenized region using primers CX3UTR2FS and CX3UTR22R2.

### Luciferase Reporter Assay

Wild-type (pmirCX3UTRWT) and mutant (pmirCX3UTRMT) plasmids containing the 3′UTR of the CX_3_CR1 mRNA, as well as the parental pmirGLO vector (Promega), were used in cotransfection experiments with the mirVana miRNA Mimic miR-27a-5p and the mirVana miRNA Mimic Negative Control #1 (Ambion). HEK293T cells were used for cotransfection experiments. The day before transfection 8 × 10^4^ cells per well were plated in 24-well plates in 500 µL of DMEM supplemented with 10% FCS. Cells were transfected with 100 ng of plasmid and 20 pmol of mimic miRNA using lipofectamine 2000 (Invitrogen) according to the manufacturer protocol. Twenty-four hours post transfection cells were harvested. Firefly and Renilla Luciferase activities were determined on cell lysates using the Dual Luciferase Reporter Assay System (Promega) according to the manufacturer guidelines. A single-tube DLReady validated luminometer TD-20/20 Turner Biosystems was used. Firefly luciferase activity was normalized to renilla luciferase activity. Normalized values were expressed as changes relative to the value of the negative control, which was set as 1. Three independent experiments were performed in quadruplicate.

### miR-23a-27a-24-2 Cluster Primary Transcript Expression

The 1 × 10^6^ NK cells from healthy donors were cultured in the presence or in the absence of 5 ng/mL of TGF-β1. Real-time PCR was performed using the hsa-mir-23a TaqMan Pri-miRNA Assay (Applied Biosystems). Hsa-mir-23a pri-miRNA expression was normalized to the GAPDH expression. Experiments were performed in triplicate.

### Transient Transfection of NK Cells

The 1 × 10^6^ NK cells from healthy donors were cultured in 24-well plates in 500 µL RPMI + 10% serum in the presence of TGF-β1 (5 ng/mL). Cells were transfected with 20 pmol of miR-27a-5p inhibitor (Applied Biosystems) or with a negative control (mirVana™ miRNA Inhibitor, Negative Control #1; Ambion) using Lipofectamine 3000 according to manufacturer instructions. Efficiency of transfection, determined with a fluorescently labeled miRNA (Cy3™ Dye-Labeled Pre-miR Negative Control #1; Ambion) was about 30% (data not shown). Cells were harvested after 72 h, RNA was extracted, and CX_3_CR1 mRNA levels were determined as described previously.

### CX3CR1-Lentiviral Vector Generation

Primer CX3ATGKZF, designed to contain a Kozak consensus sequence, and primer CX3UTR2R (Figure S1 in Supplementary Material) were used for the amplification of a genomic fragment (2,494 bp in length) containing the whole CX3CR1 gene open reading frame (ORF) and a portion of the 3′UTR in which the two putative target sites for miR-27a-5p are included. The product was cloned in a pcDNA 3.1/V5-His-TOPO vector (Invitrogen), excised by XbaI-BamHI digestion, and ligated to an XbaI-BamHI digested pCDH-CMV-MCS-EF1-GFP lentiviral vector (System Biosciences).

Ligation product was transformed in Stbl3 *E. coli* cells (Invitrogen), a positive colony was selected by PCR, endotoxin-free plasmid DNA was extracted using the QIAfilter Plasmid Midi Kit with the EndoFree Plasmid Buffer Set (Qiagen), the whole insert and the flanking vector regions were sequenced using 16 primers (whose sequences are reported in Figure S1 in Supplementary Material).

### Virus Packaging

The lentiviral vector containing the CX_3_CR1 gene was assembled in lentiviral particles using the pPACKH1 HIV Lentivector Packaging Kit (System Biosciences). Briefly, 3 × 10^6^ 293T cells plated in a 10 cm plate were tranfected with the packaging plasmids and the CX_3_CR1 lentiviral vector with lipofectamine 2000 (Invitrogen). Medium was changed every 24 h and collected at 48 and 72 h. Virus containing supernatants were pooled and concentrated by the use of a PEG precipitation solution. Titration was performed by flow cytometric detection of GFP fluorescence emitted by infected cells.

### HEK293T Cells Transduction

HEK293T cells (2 × 10^5^) were plated in six-well plates and infected with viral preparation at a multiplicity of infection of 2 in 500 µL complete medium without antibiotics in the presence of 8 µg/mL of polybrene (Santa Cruz Biotechnology, Dallas, TX, USA). Infected cells were centrifuged for 1 h at room temperature at 2,000 rpm, then incubated at 37°C. 24 h after infection, medium was removed and replaced with 4 mL of fresh medium without polybrene. One week after infection, CX_3_CR1 positive cells were selected using the anti-CX_3_CR1 Microbead Kit (Miltenyi Biotec). Clones generated by limited dilution of the selected cells were analyzed for CX_3_CR1 surface expression by flow cytometry. Clones showing a good and stable expression of CX_3_CR1 were expanded and used for transfection with miR-27a-5p and with miR-Neg.

### Transfection of CX_3_CR1-Expressing HEK293T Cells

An HEK293T clone stably expressing CX_3_CR1 was transfected with miR-27a-5p and with miR-Neg to evaluate the modulation of CX_3_CR1 expression. The 10^5^ cells were plated in six-well plates in 2 mL complete medium, transfection was performed using 100 pmol of miRNA mimics and 3 μL of Lipofectamine RNAiMAX (Invitrogen) according to the manufacturer guidelines. Cells were harvested 48 and 72 h after transfection. CX_3_CR1 expression was analyzed by flow cytometry.

### Coculture of NK Cells and SH-SY5Y NB Cell Line in Transwell Condition

For coculture experiments in transwell condition, 2 × 10^5^ resting NK cells were cultured for 48 h with 3 × 10^5^ SH-SY5Y cells. NK cells and SH-SY5Y cells were placed in 24-well transwell (0.3-µm pore size; Corning Costar), upper and bottom chamber, respectively (12).

### Statistical Analysis

The statistical level of significance (*p*) is indicated using Student’s *t*-test, a parametric significance test. Graphic representation and statistical analysis were performed using GraphPad Prism 6 (GraphPad Software, La Jolla, CA, USA). Pearson’s correlation coefficient (two-tailed test) was used to evaluate the correlation between CX_3_CR1 mRNA and miR-27a-5p expression.

## Results

### miRNA Profiling of TGF-β1-Treated NK Cells

**Figure 1:**
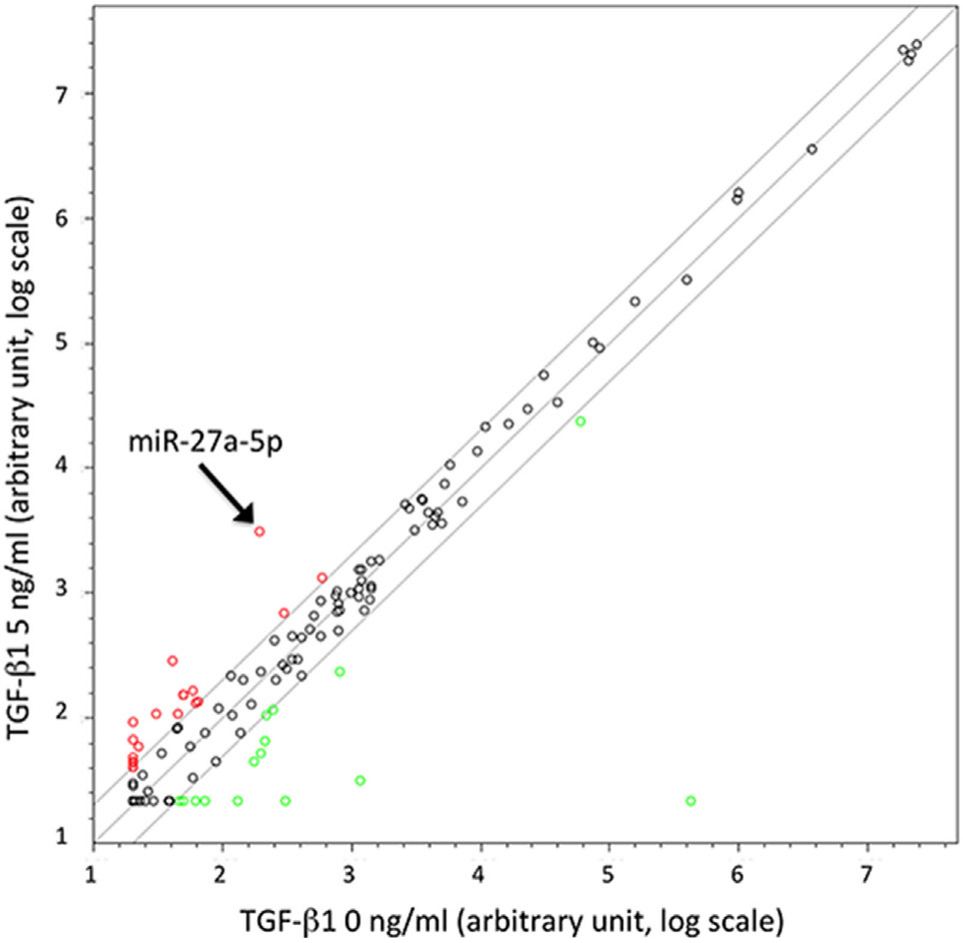
Scatter plot showing microRNAs (miRNAs) up- or downregulated in TGF-β1-treated natural killer (NK) cells. Results are referred to NK cells from a representative healthy donor of three analyzed (donors 1, 2, and 3) treated with TGF-β1 (5 ng/mL). Plot is referred to TaqMan Array Human MicroRNA Cards B (Applied Biosystems). Results are expressed as arbitrary units on a log scale. U6 snRNA has been used as reference control. miR-27a-5p is indicated by the arrow. Diagonal lines represent the 2, 1, and 0.5 values for fold induction.

To identify miRNAs possibly involved in the TGF-β1-mediated modulation of chemokine receptors expression, we investigated the miRNA expression profile variation in TGF-β1-treated NK cells purified from the peripheral blood of three unrelated healthy donors. The analysis, allowing the simultaneous profiling of 754 miRNAs (Figure 1), showed that miRNAs up- or downregulated by the treatment ranged from 127 in donor 2 to 234 in donor 3 (Figure S2 in Supplementary Material). A total of 14 miRNAs were modulated in NK cells from all donors analyzed upon treatment with either 5 or 40 ng/mL of TGF-β1. Among these 14 miRNAs, 11 were excluded from further analysis being expressed at low levels (threshold cycle <33), while 1 (miR-1201) was forsaken due to its sequence overlapping to an annotated small nucleolar RNAs (snoRNA, SNORD126), as reported in miRBase.^8^ The remaining two miRNAs (miR-302a and miR-27a-5p) were further investigated as putative regulators of CXCR4, CXCR3, or CX_3_CR1 expression (Figure 1 and Figure S2 in Supplementary Material).

### miR-27a-5p As Putative Regulator of CX_3_CR1 Expression

**Figure 2:**
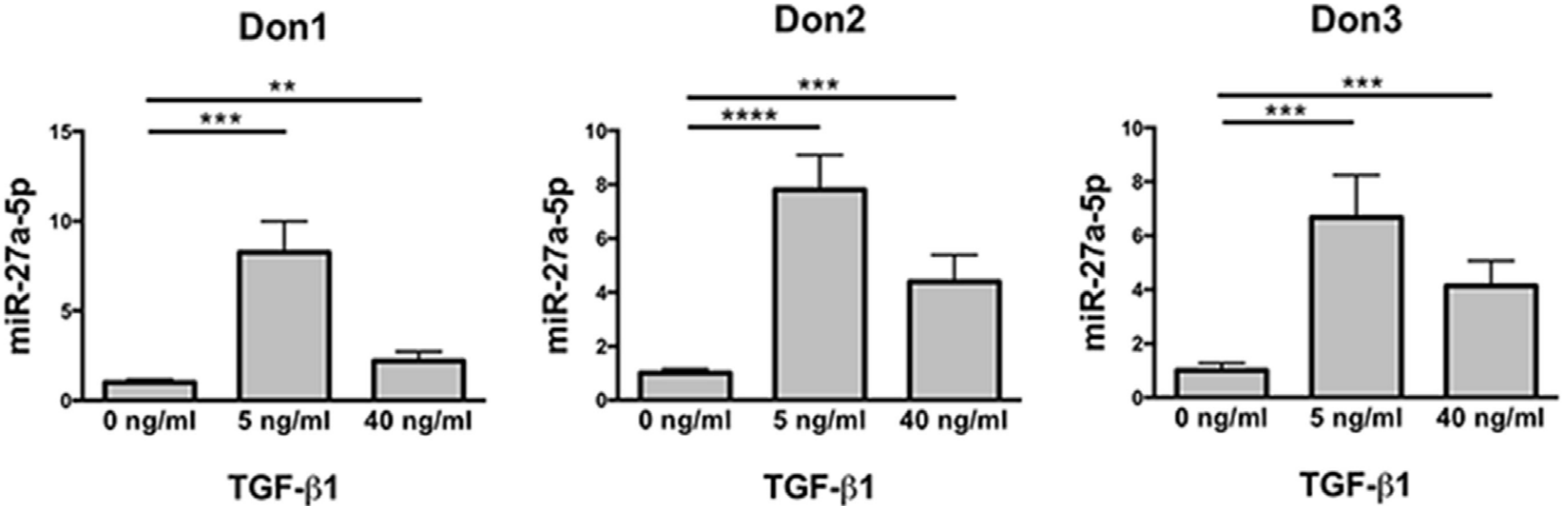
Expression profile of miR-27a-5p in TGF-β1-treated natural killer (NK) cells. NK cells untreated or treated for 24 h with the indicated concentration of TGF-β1 were analyzed for miR-27a-5p expression. RNU44 was used as reference control. Experiments were performed in quadruplicate on NK cells derived from three healthy donors (donors 1, 2, and 3). Expression relative fold changes are referred to the expression of untreated cells, whose miR-27a-5p expression has been arbitrarily assigned the value 1. **p < 0.01, ***p < 0.001, and ****p < 0.0001.

The differential expression of miR-302a and miR-27a-5p in untreated and TGF-β1-treated NK cells was checked for validation by using specific miRNA assays. The analysis revealed that miR-302a was virtually absent in both untreated and TGF-β1-treated NK cells (data not shown). On the contrary, in agreement with data obtained by miRNA profiling, TGF-β1-treated NK cells showed a significant increase of miR-27a-5p expression, which was higher at low TGF-β1 concentration (Figure 2). Thus, miR-27a-5p was further investigated as putative regulator of CXCR4, CXCR3, or CX_3_CR1 expression using the computational prediction on-line tools TargetScan (see text footnote 3) (23), miRanda (see text footnote 4) (24), and miRmap (see text footnote 5) (25). The analysis indicated miR-27a-5p as a putative regulator of CX_3_CR1 expression with relatively high scores. The three softwares predicted the same principal site of interaction in the CX_3_CR1 3′UTR with the miR-27a-5p seed region (Figure S3A in Supplementary Material). An additional site in the CX_3_CR1 3′UTR, with a weaker interaction with miR-27a-5p, was predicted by miRanda and miRmap tools only (Figure S3B in Supplementary Material).

### Inverse Correlation of miR-27a-5p and CX_3_CR1 mRNA Expression in TGF-β1-Treated NK Cells

**Figure 3:**
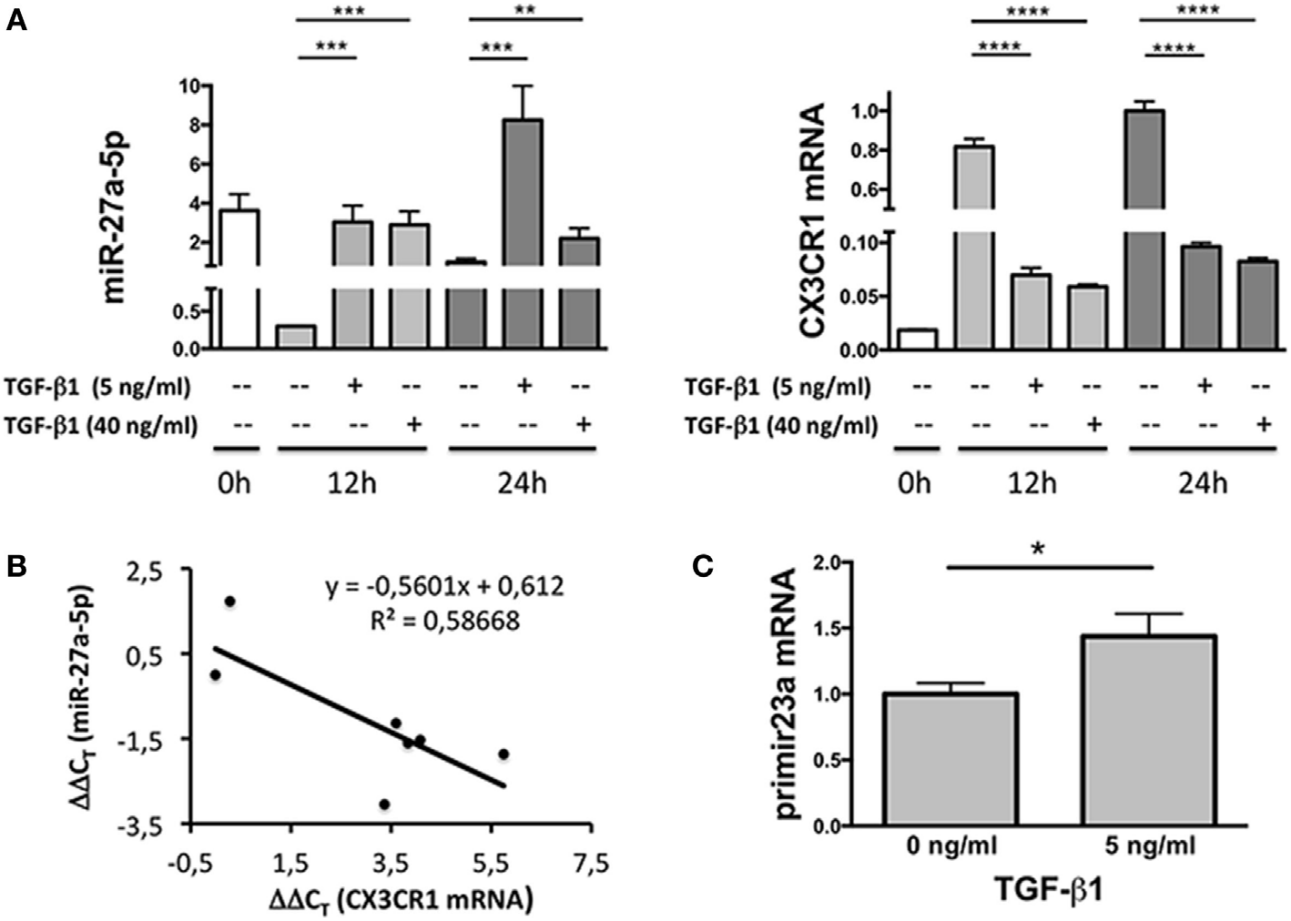
Expression profile of miR-27a-5p and CX_3_CR1 mRNA in TGF-β1-treated natural killer (NK) cells. **(A)** NK cells treated for 12 or 24 h with the indicated amounts of TGF-β1 were simultaneously analyzed for the expression of miR-27a-5p and CX_3_CR1. RNU44 and GAPDH were used as reference controls, respectively. Data in quadruplicate from one representative donor (donor 1), of two analyzed (donors 1 and 4), are shown. Expression relative fold changes are referred to the expression of miR-27a-5p and CX_3_CR1 mRNA in untreated NK cells (medium alone, 24 h) whose expression has been arbitrarily assigned the value 1. **p < 0.01, ***p < 0.001, ****p < 0.0001. **(B)** Correlation between miR-27a-5p and CX_3_CR1 mRNA expression in TGF-β1-treated NK cells. Scatter plot showing miR-27a-5p and CX_3_CR1 mRNA expression in TGF-β1-treated NK cells from the same donor (donor 1) of (**A**). Each point represents the miR-27a-5p and CX_3_CR1 expression detected in one of the seven reported conditions, with different TGF-β1 concentrations (0, 5, and 40 ng/mL) and time of treatment (0, 12, and 24 h). The trendline, its equation and the *R*^2^ value is reported. Significance of correlation was determined using the Pearson’s coefficient (*r* = −0.765948064, *p* < 0.05). Data are referred to a representative donor (donor 1), of two analyzed (donors 1 and 4). **(C)** miR-23a-27a-24-2 cluster primary transcript expression in TGF-β1-treated NK cells. NK cells cultured for 24 h in the presence or absence of 5 ng/mL of TGF-β1 were analyzed by real-time PCR for the expression of miR-23a~27a~24-2 cluster primary transcript. GAPDH was used as reference control. Data in triplicate from one representative donor of three analyzed (donors 5, 6, and 7) are shown. Expression relative fold changes are referred to the expression of untreated NK cells (medium alone) whose expression has been arbitrarily assigned the value 1. *p < 0.05.

Since miRNAs properties include the capability of inducing degradation of targeted mRNAs, expression profiles of miR-27a-5p and CX_3_CR1 mRNAs were simultaneously analyzed over time. As shown in Figure 3A, at both concentrations used, TGF-β1 caused a significant increment of miR-27a-5p and decrease of CX_3_CR1 mRNA expression. Importantly, expression of miR-27a-5p and CX_3_CR1 mRNA was inversely correlated (Pearson’s correlation coefficient *r* = 0.766, *p* < 0.05; Figure 3B). While preliminary data showed that 1 ng/mL of TGF-β1 may be sufficient for induction of miR-27a-5p, concentrations of 5 and 40 ng/mL were chosen for a more systematic investigation. Next, to deepen the molecular mechanism responsible for the TGF-β1-induced increase of miR-27a-5p, we analyzed the expression of the miR-23a-27a-24-2 cluster, precursor of miRNAs, including miR-27a-5p. As shown in Figure 3C, TGF-β1 caused a significant increment of the primary miR-23a-27a-24-2 transcript, thus demonstrating that the increased amount of miR-27a-5p might be due, at least in part, to induction of its gene expression other than, for example, to miR-27a-5p egress from intracellular stores.

### miR-27a-5p Interacts with the CX_3_CR1 mRNA

**Figure 4:**
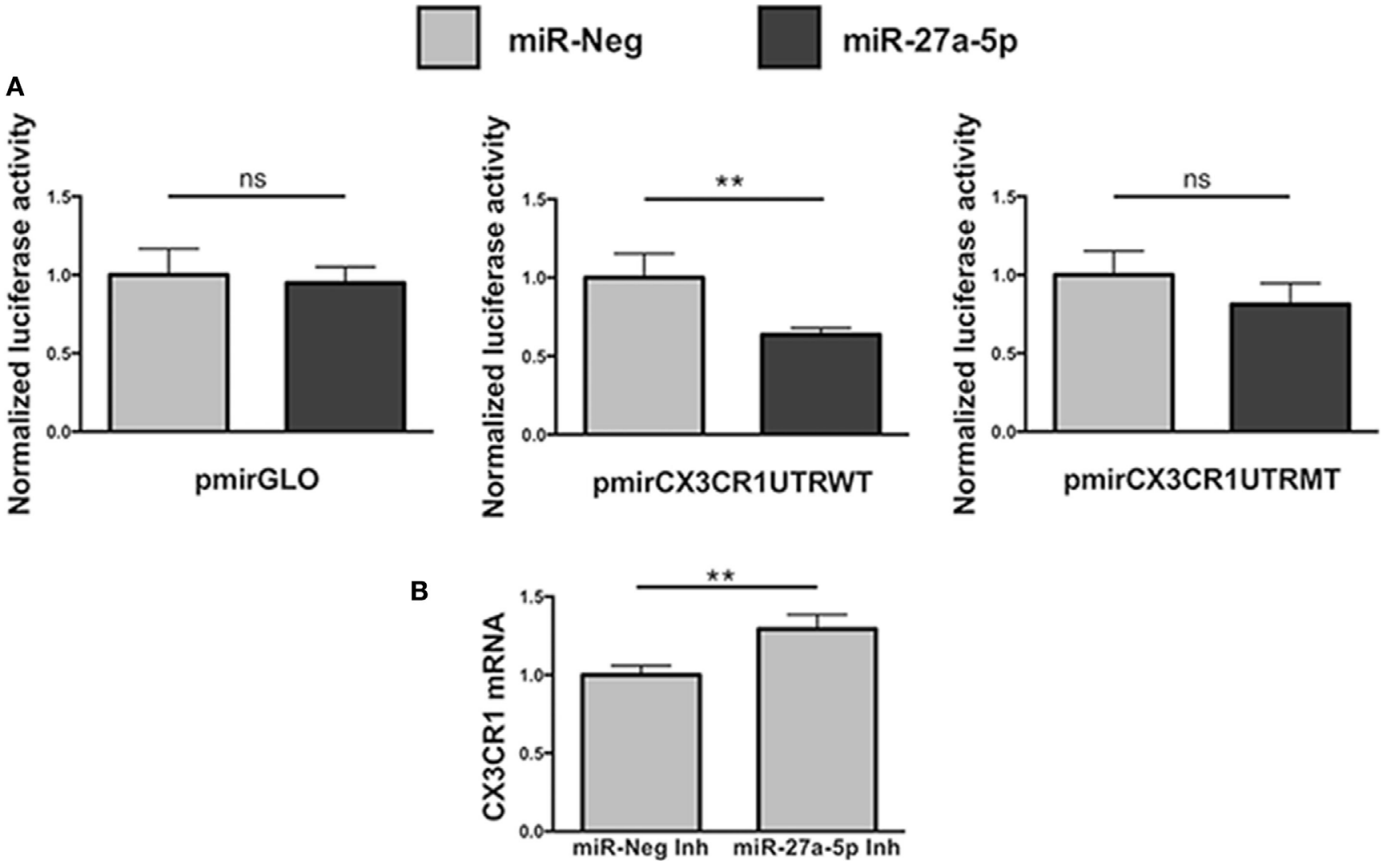
Functional interaction between miR-27a-5p and CX_3_CR1 mRNA. **(A)** Luciferase activity in HEK293T cells cotransfected with luciferase reporter construct containing the 3′ untranslated region (3′UTR) CX_3_CR1 and miR-27a-5p mimics. Firefly luciferase activity was normalized to the renilla luciferase activity, expressed by the same vector. Data in quadruplicate are from one experiment of three performed. Luciferase activities are referred to the activity of cells transfected with the miRNA negative control whose expression has been arbitrarily assigned the value 1. pmirGLO, parental vector; pmirCX3CR1UTRWT, pmirGLO vector containing 700 bp of the CX_3_CR1 3′UTR downstream of the firefly luciferase gene; pmirCX3CR1UTRMT, mutated version of pmirCX3CR1UTRWT containing the C>G mutation in the CX_3_CR1 3′UTR target site; miR-Neg, miRNA mimic negative control. **p < 0.01; ns, not significant. **(B)** CX_3_CR1 mRNA expression in NK cells transfected with miR-27a-5p inhibitor (Inh) or with a negative control miRNA Inh. Cells were cultured for 72 h in the presence of 5 ng/mL of TGF-β1. Data in triplicate are from one representative healthy donor of three analyzed (donors 8, 9, and 10). GAPDH has been used as reference control. Expression relative fold changes are referred to CX_3_CR1 mRNA expression in NK cells transfected with the negative control miRNA inhibitor, whom expression has been arbitrarily assigned the value 1. *p < 0.05.

To unequivocally demonstrate that miR-27a-5p could interact with the 3′UTR of the CX_3_CR1 mRNA, modulating its expression, we performed a luciferase reporter assay (Figure 4). We cloned a 700 bp fragment of the CX_3_CR1 gene 3′UTR, containing all the putative sites of interaction with the seed region of miR-27a-5p, into a luciferase reporter vector, downstream of the firefly luciferase gene. Moreover, we also produced a mutated version of the construct, containing a C>G substitution in the main site of the CX_3_CR1 3′UTR (Figure S3A in Supplementary Material). The two plasmids were separately cotransfected in HEK293T cells with a miR-27a-5p mimic or with a random sequence miRNA negative control. As shown in Figure 4A, a significant reduction of the normalized luciferase activity was detected in the presence of miR-27a-5p mimic compared to the miRNA negative control. Importantly, this effect was lost when the miR-27a-5p mimic was cotransfected with the construct having the mutated sequence of the CX_3_CR1 3′UTR, thus confirming the interaction of miR-27a-5p with the CX_3_CR1 3′UTR.

Next, to further confirm miR-27a-5p and CX_3_CR1 mRNA interaction, before TGF-β1 treatment NK cells were transiently transfected with a specific miR-27a-5p inhibitor or with a scrambled miRNA as negative control (Figure 4B). As expected, we observed a significantly higher CX_3_CR1 mRNA expression in TGF-β1-treated NK cells previously transfected with the miR-27a-5p inhibitor when compared with cells transfected with a scrambled miRNA.

### miR-27a-5p Downregulates the Surface Expression of CX_3_CR1

**Figure 5:**
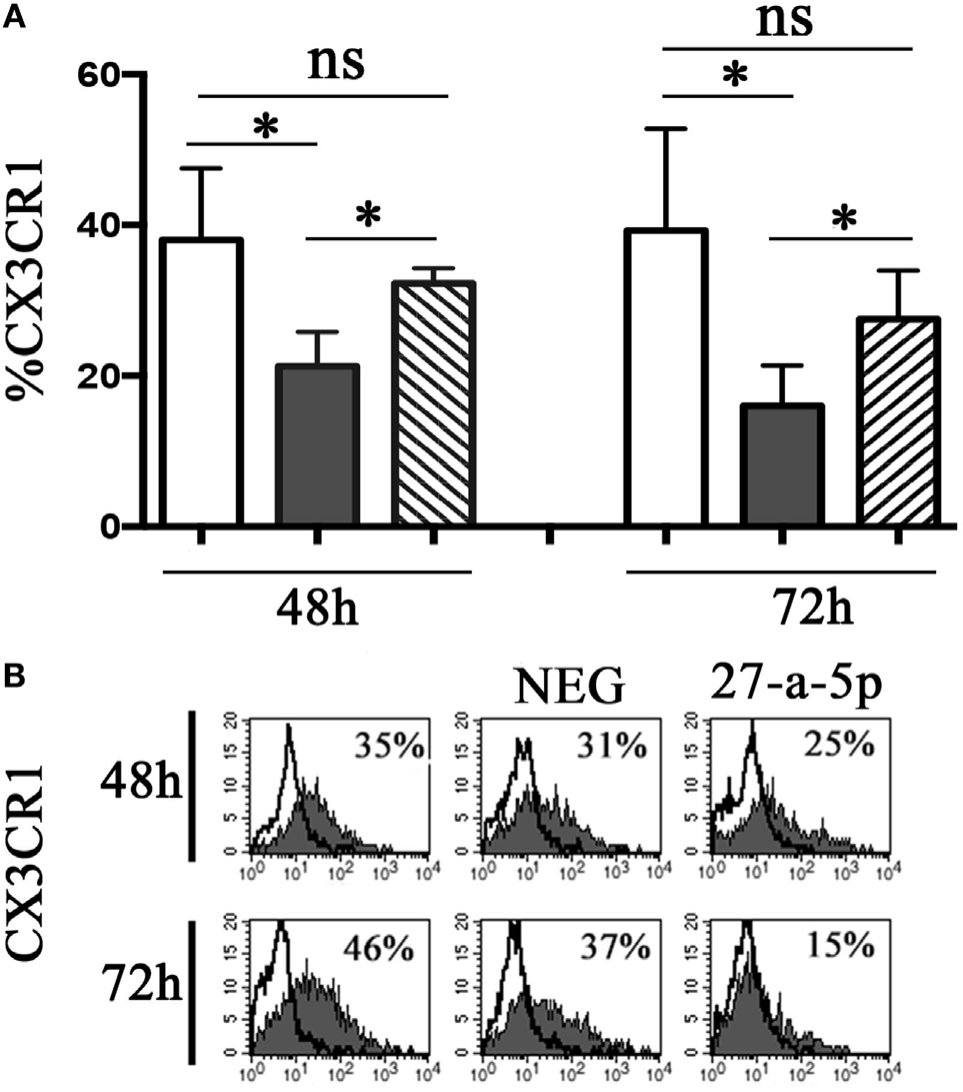
miR-27a-5p-induced downregulation of CX_3_CR1 surface expression. **(A)** CX_3_CR1^+^ HEK293T cells (clone #124) untrasfected (white bar), transfected with miR-27a-5p mimic (black bar), or miRNA negative control (stripped bar) were analyzed by flow cytometry for the expression of CX_3_CR1 at the indicated time intervals. Average of four independent experiments. 95% confidence intervals and significance (*p < 0.05) are shown. ns, non-significant. **(B)** Representative cytofluorimetric analysis of CX_3_CR1 surface expression. Values inside each histogram indicate the percentage (%) of positive cells. NEG: miRNA negative control.

To determine whether miR-27a-5p is able to downregulate the surface expression of CX_3_CR1, we prepared a lentiviral vector containing the whole ORF of the CX_3_CR1 gene and the portion of the 3′UTR region containing the two putative binding sites for miR-27a-5p. HEK293T cells where transduced with the vector and cultured under limiting dilution to obtain CX_3_CR1 positive clones. A clone (#124) was selected that stably expressed at the cell surface optimal levels of the chemokine receptor. Clone #124 was transfected with a miR-27a-5p mimic or with a miRNA negative control and analyzed by flow cytometry for the chemokine receptor expression. As shown in Figure 5, miR-27a-5p mimic induced a significant downregulation of the percentage of CX_3_CR1 positive cells. On the contrary, the expression of other molecules such as PVR (CD155) was unaffected (Figure S4 in Supplementary Material). These data further support the role of miR-27a-5p as modulator of the chemokine receptor.

### miR-27a-5p Induction in the Context of NB-NK Cocultures

**Figure 6:**
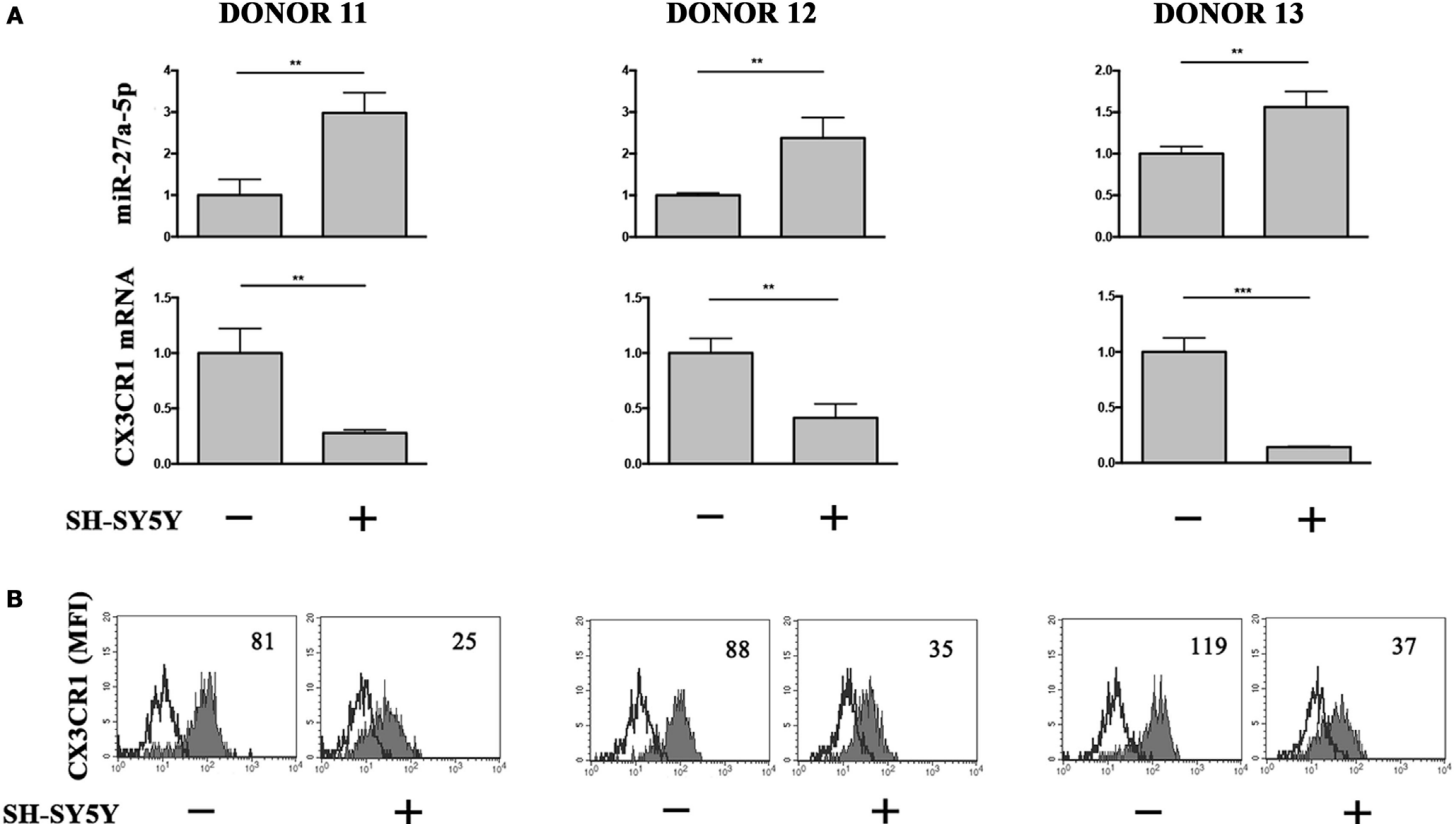
miR-27a-5p and CX_3_CR1 expression in NK cells cocultured under transwell conditions with SH-SY5Y neuroblastoma cell line. **(A)** miR-27a-5p and CX_3_CR1 mRNA expression of SH-SY5Y-conditioned NK cells from three healthy donors (donors 11, 12, and 13). Data in triplicate from each donor are shown. RNU44 and GAPDH were used as reference controls, respectively. **p < 0.01; ***p < 0.001. **(B)** Cytofluorimetric analysis of CX_3_CR1 expression in NK cells from the same donors. White profiles refer to cells incubated with isotype-matched mAbs. Value inside each histogram indicates the median fluorescence intensity (MFI).

To analyze the relevance of miR-27a-5p induction in a pathological context, we cocultured under transwell condition resting NK cells and the prototypic SH-SY5Y NB cell line, which we described to induce a TGF-β1-mediated downregulation of CX_3_CR1 surface expression (12). Accordingly with our previous published data (12), NB conditioning resulted in a significant downregulation of CX_3_CR1 expression at both protein and mRNA level. Importantly, this modulatory effect matched with a significant increase of miR-27a-5p as compared to unconditioned NK cells (Figure 6).

## Discussion

A previous study by our group reported that TGF-β1 released by NB cells is able to modify the chemokine receptor repertoire of NK cells (12). In particular, it downregulates CX_3_CR1 expression in resting NK cells. In the present article, we show that TGF-β1-induced miR-27a-5p directly modulates the expression of CX_3_CR1 mRNA at the post-transcriptional level. Increment of miR-27a-5p expression is due to upregulation of the miR-23a-27a-24-2 cluster, which contains, among others, miR-27a-5p. Importantly, we observed that this regulatory mechanism also occurs in NK cells during NB-conditioning.

All our results were obtained using unfractioned blood NK cells from healthy donors, mostly represented (more than 90%) by the CD56^dim^ population (7, 26). Since CD56^bright^ NK cells virtually do not express CX_3_CR1, further experiments should clarify whether miR-27a-5p might contribute to their costitutive CX_3_CR1^low/neg^ phenotype.

The downregulation of CX_3_CR1 has important consequences on NK cells function, as CX_3_CR1, with other chemokine receptors, drives, at steady state, NK cell localization in peripheral tissues as well as their migration under inflammatory conditions (27). In particular, it has been shown that the relative expression of CX_3_CR1 and CXCR4 regulates NK cell migration within the BM and their egress from it (28, 29). Moreover, in a model of experimental autoimmune encephalomyelitis (EAE), CX_3_CR1/fractalkine axis specifically recruits NK cells into the brain (30). Indeed in CX_3_CR1-deficient mice with EAE the amount of NK cells in the inflamed CNS cells was markedly reduced, as compared to wild-type mice, whereas that of other cell types, including T, NKT cells, and monocyte/macrophages, did not show significant variation (30). Importantly, recruitment of CX_3_CR1+ NK cells toward the inflamed CNS ameliorated the EAE disease severity, since mature NK cells showed a higher cytolytic activity against autoreactive CD4+ T cells (21).

Interestingly, CX_3_CR1/CX_3_CL1 axis, other than regulate NK cell migration, increases NK cell responsiveness to CCL4 (MIP1β) and CXCL8 (IL-8) (31) and promotes IFN-γ production and cytotoxicity by NK cells (32).

The functional relevance of CX_3_CR1 in NK cells tropism and activity is further underlined by the identification of viruses producing viral chemokines able to bind CX_3_CR1: vMIP-II, encoded by the Kaposi’s sarcoma-associated herpesvirus, which inhibits naive migration of CD56^dim^ NK cells blocking the binding of CX_3_CL1 (33), and vCXCL1, encoded by human cytomegalovirus, which induces NK and neutrophils migration, presumably to facilitate a neutrophil-mediated viral dissemination (34).

CX_3_CR1 expression also has a pivotal role in tumors, conditioning migration and adhesion of tumor cells, and tumor invasiveness and metastasis (35). In particular, CX_3_CR1 expression in pancreatic and prostate cancer cells increases invasiveness and metastasis to neuronal tissues (36). Interestingly, however, while CX_3_CR1 expression in cancer is generally associated with invasiveness and metastasis, the coexpression of CX_3_CR1 and CX_3_CL1 by the same cell type, as occurs in human colorectal cancer cells, acts as a retention factor, limiting tumor spreading to metastatic sites (37).

Due to the wide properties of CX_3_CR1, we can argue that the receptor downregulation caused by TGF-β1 in mature cytolytic NK cells might severely hamper their recruitment and functions at tumor sites. This might occur thanks the TGF-β1-induced upregulation of the miR-23a-27a-24-2 cluster in NK cells, which in turn causes miR-27a-5p upregulation and the consequent CX_3_CR1 downregulation. It is likely that a tight control of the miR-23a-27a-24-2 cluster expression is necessary, given that its deregulation has been reported in several tumors and other diseases (38). Interestingly, TGF-β1 has been shown to be responsible for upregulation of the miR-23a-27a-24-2 cluster in hepatocellular carcinoma cells (39), in lung adenocarcinoma cells (40) as well as in CD8 T cells (41). In these lymphocytes, additionally, upregulation of the cluster has been associated with inhibition of IFN-γ expression and reduced cytotoxicity (41). miR-27a-5p has been also reported to downregulate perforine 1 (Prf1) and granzyme B (GzmB) expression in resting and IL-15-activated NK cells (42), thus hampering NK cells cytotoxicity. NK92 and primary NK cells overexpressing miR-27a-5p showed a reduced cytotoxicity but unmodified levels of the activating receptors NKG2D, NKp30, NKp44, and NKp46. Moreover, knockdown of miR-27a-5p in NK cells increased *in vitro* cytotoxicity and decreased tumor growth in a human tumor xenograft model (42). Overall, these data suggest that miR-23a-27a-24-2 cluster could be an important target of TGF-β1, possibly acting as an intermediate inducer of its effects. miR-27a-5p, a product of this cluster, is able to regulate the expression of multiple targets crucial for NK cells function. Thus, it appears as a key node for NK activity control, being able to dampen NK cell recruitment, cytotoxicity, and IFN-γ production, potentially representing, in perspective, an interesting cancer therapeutic target.

## Author Contributions

SR, CB, and RC designed the study. SR, FC, AD, BC, FR, FL, and RC performed the experiments, analyzed, and interpreted data. SR, FC, AM, CB, and RC wrote the manuscript. All the authors read and approved the final article.

## Conflict of Interest Statement

AM is a founder and shareholder of Innate-Pharma (Marseille, France). The remaining authors declare no conflicts of interest.

## Footnotes

^1^http://www.sabiosciences.com/mirnaArrayDataAnalysis.php.

^2^http://www.ncbi.nlm.nih.gov/geo/.

^3^www.targetscan.org.

^4^www.microrna.org.

^5^http://mirmap.ezlab.org.

^6^http://www.ncbi.nlm.nih.gov/tools/primer-blast/.

^7^http://www.genomics.agilent.com.

^8^www.mirbase.org.
