# Supplementary figures and images for: TGF-β1 Downregulates the Expression of CX3CR1 by Inducing miR-27a-5p in Primary Human NK Cells
Source: Front Immunol. 2017 Jul 25;8:868. doi: 10.3389/fimmu.2017.00868 (PMC5524732; doi:10.3389/fimmu.2017.00868)

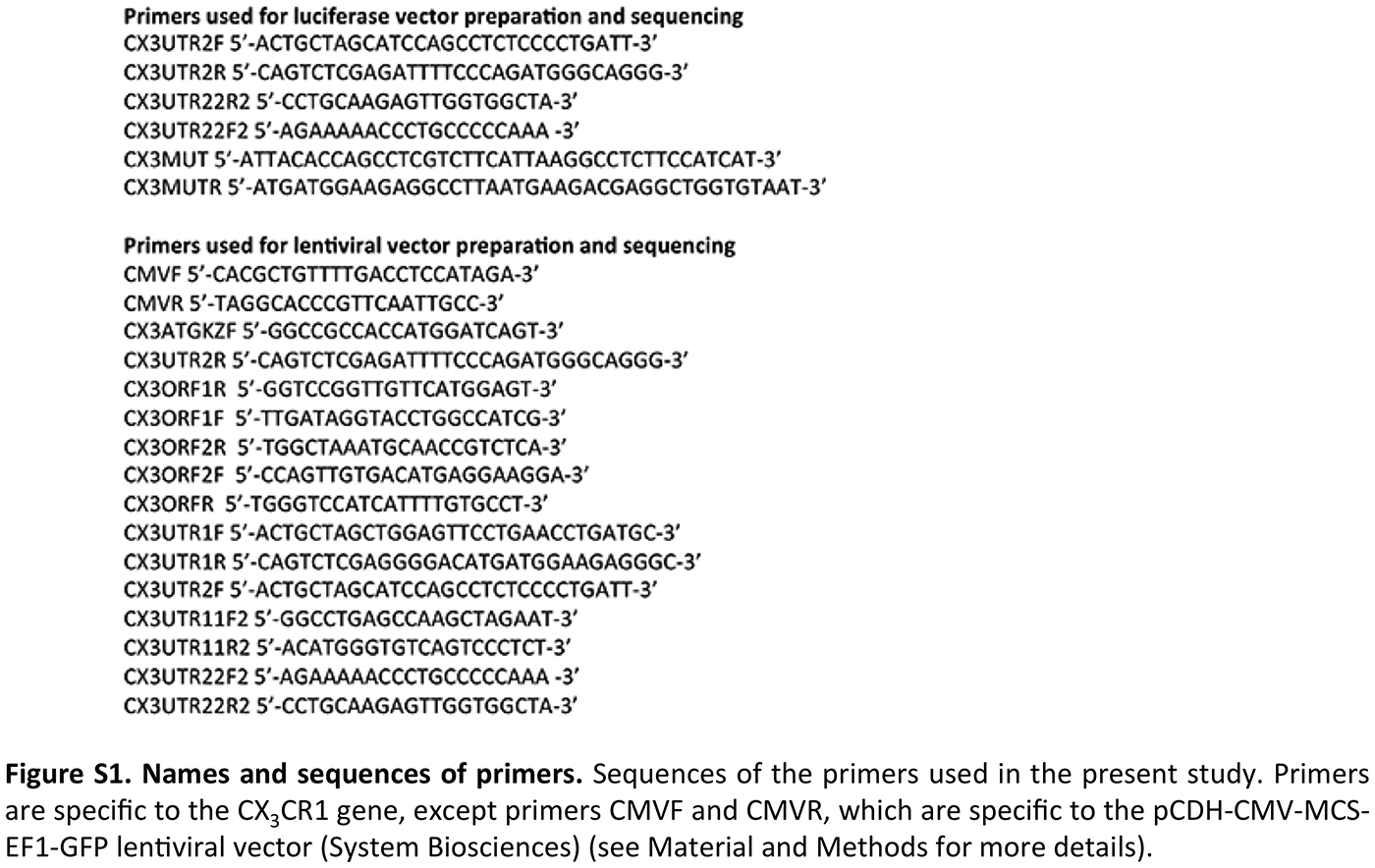

Supplement: Supplementary file 1 [file Image_1.TIF]

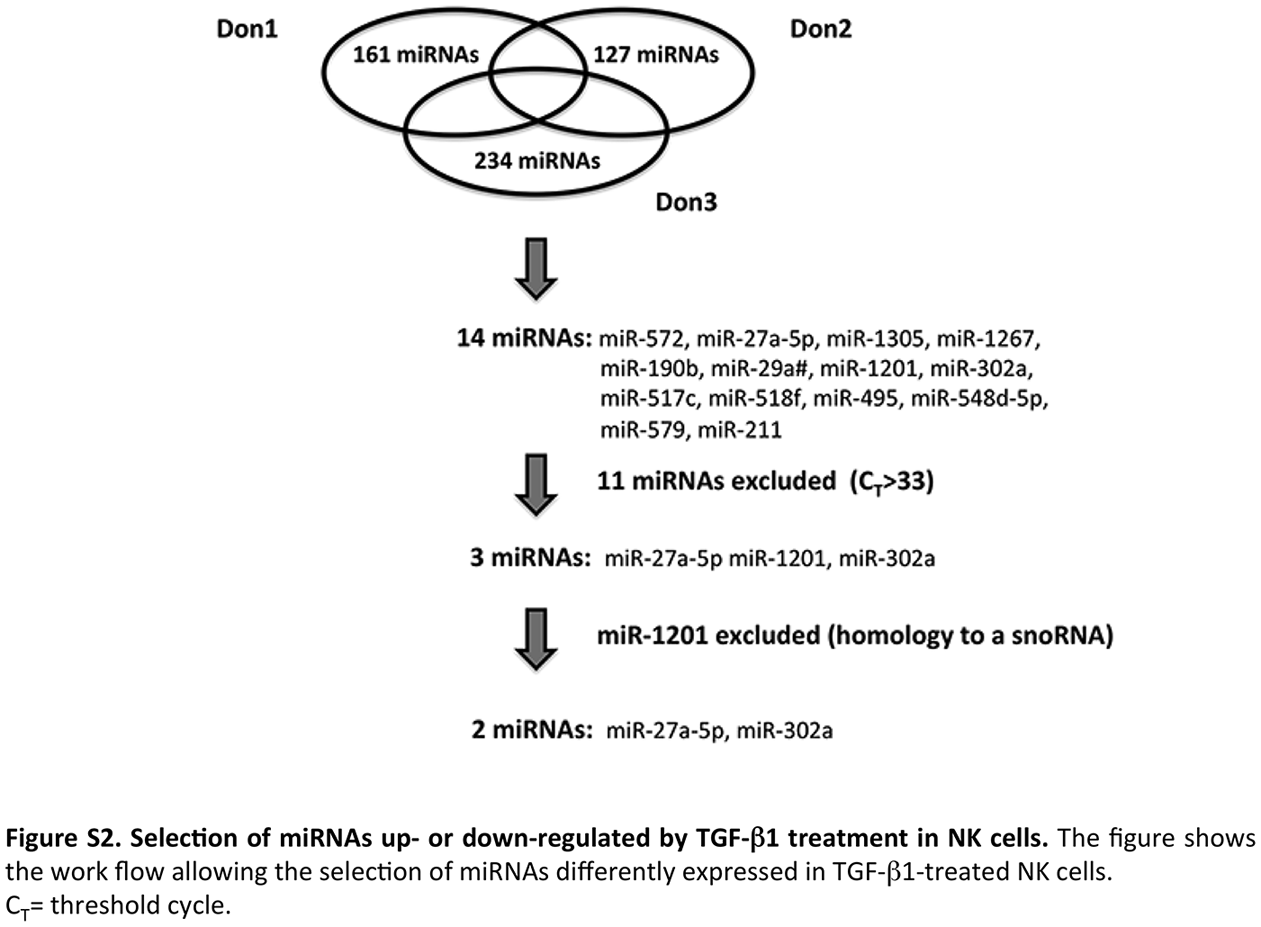

Supplement: Supplementary file 2 [file Image_2.TIF]

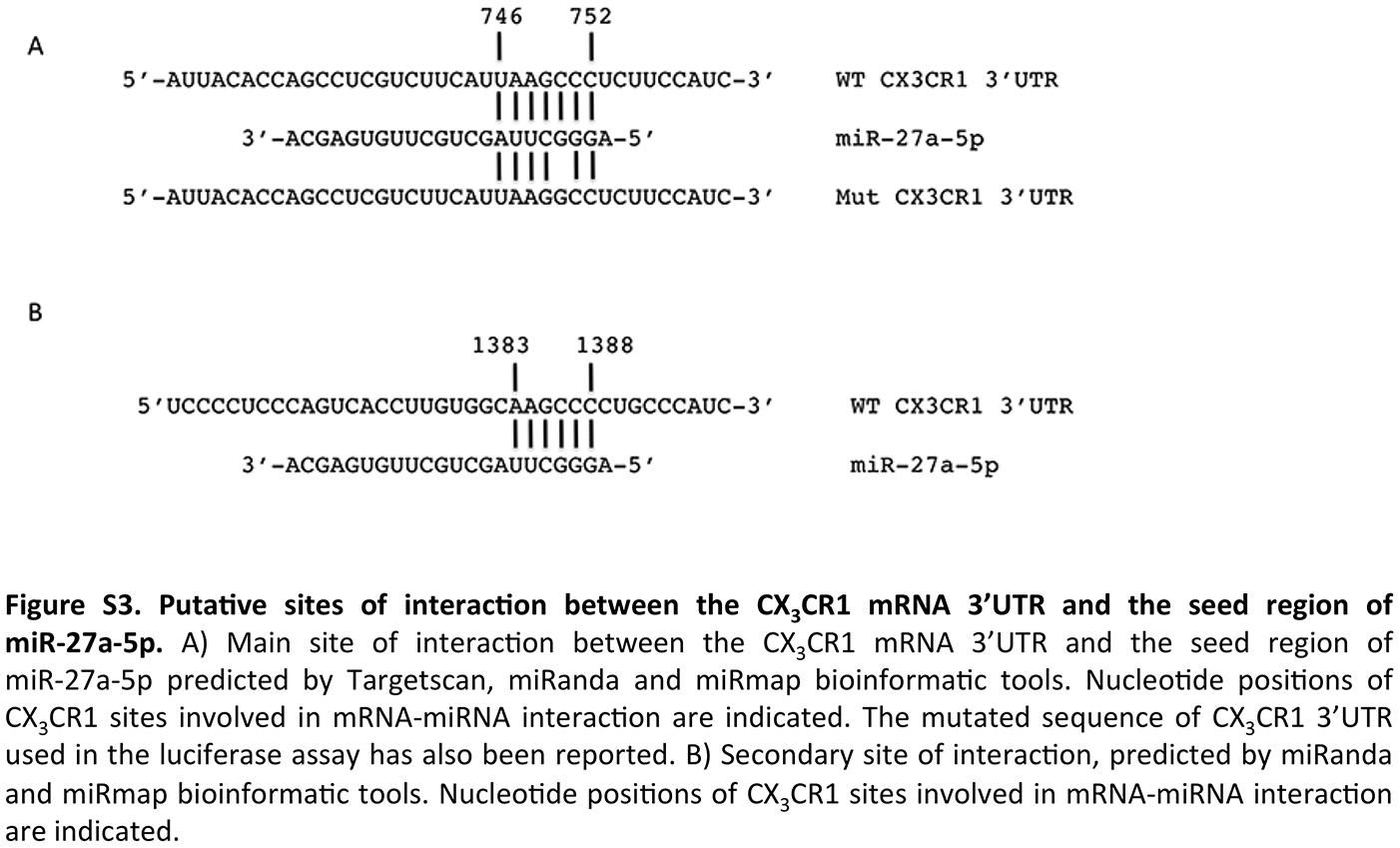

Supplement: Supplementary file 3 [file Image_3.TIF]

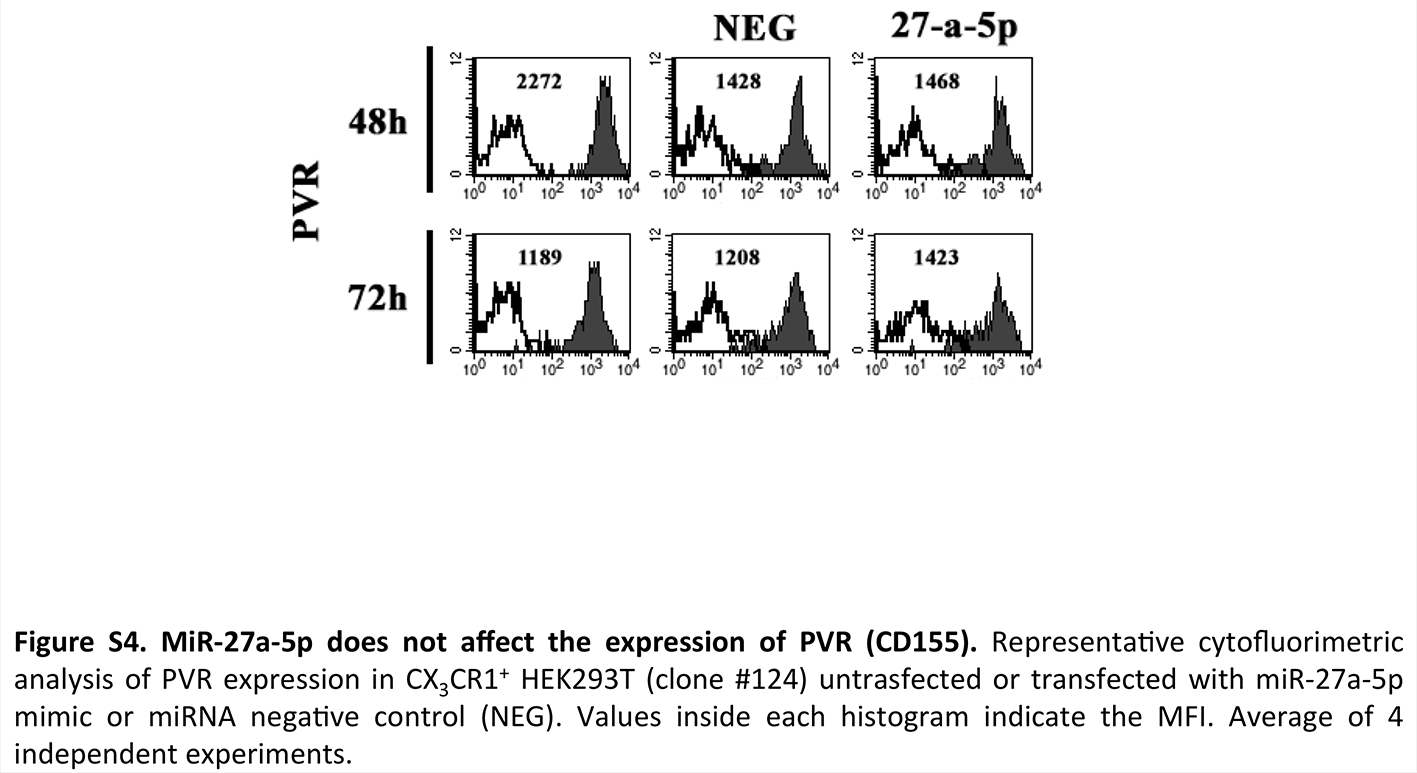

Supplement: Supplementary file 4 [file Image_4.TIF]
